# Analysis of Contractility and Invasion Potential of Two Canine Mammary Tumor Cell Lines

**DOI:** 10.3389/fvets.2017.00149

**Published:** 2017-09-12

**Authors:** Kaisa Rajakylä, Ramaswamy Krishnan, Sari Tojkander

**Affiliations:** ^1^Faculty of Veterinary Medicine, Department of Veterinary Biosciences, Section of Pathology, University of Helsinki, Helsinki, Finland; ^2^Beth Israel Deaconess Medical Center, Harvard Medical School, Boston, MA, United States

**Keywords:** canine mammary tumor, breast cancer, actin cytoskeleton, invasion, contractility, actomyosin forces, AMP-activated protein kinase, traction force imaging

## Abstract

Cancer cells are surrounded by a mechanically and biochemically distinct microenvironment that undergoes dynamic changes throughout the neoplastic progression. During this progression, some cancer cells acquire abnormal characteristics that potentiate their escape from the primary tumor site, to establish secondary tumors in distant organs. Recent studies with several human cancer cell lines have shown that the altered physical properties of tumor cells, such as their ability to apply high traction forces to the surroundings, are directly linked with their potential to invade and metastasize. To test the hypothetical interconnection between actomyosin-mediated traction forces and invasion potential within 3D-microenvironment, we utilized two canine mammary tumor cell lines with different contractile properties. These cell lines, canine mammary tumor (CMT)-U27 and CMT-U309, were found to have distinct expression patterns of lineage-specific markers and organization of actin-based structures. In particular, CMT-U309 carcinoma cells were typified by thick contractile actomyosin bundles that exerted high forces to their environment, as measured by traction force microscopy. These high contractile forces also correlated with the prominent invasiveness of the CMT-U309 cell line. Furthermore, we found high contractility and 3D-invasion potential to be dependent on the activity of 5′AMP-activated protein kinase (AMPK), as blocking AMPK signaling was found to reverse both of these features. Taken together, our findings implicate that actomyosin forces correlate with the invasion potential of the studied cell lines.

## Introduction

Progression of breast cancer into a metastatic disease is a major clinical problem. In normal mammary gland, the integrity of the epithelia is maintained by the contractile actomyosin bundles that provide tension for the integration of cellular junctions (1). However, during cancer progression, changes in the biophysical properties of the tumor stroma or defects in the mechanosensitive pathways of the epithelial cells may lead to deregulation of these actomyosin bundles and subsequently induce alterations in the intracellular forces (2). As cells start to exert abnormal forces, epithelial integrity may be compromised, allowing the escape of transformed cells from the primary tumor site. Besides the role of actomyosin forces in the maintenance of normal cell–cell junctions, actomyosin bundles provide traction forces through the integrin-based adhesions that enable cells to grip to the underlying substrate, migrate, and modify the environment (3). During invasion, the transformed cells rely on these actomyosin forces in penetrating the surrounding tissue and the potential to invade has thus been directly linked with abnormally high cellular forces (4–8).

While most of the cancer cell invasion studies have been performed by utilizing either human cell lines or induced mouse tumors, canine tumors have become an attractive alternative and natural model to study many carcinomas due to the clear histopathological similarities in comparison with human cancers (9–11). Canine mammary tumors (CMTs) are the most frequent neoplasms of the female dogs, about three times more common than breast cancer in women and provide a comparative model for the heterogeneous group of human breast cancers (12–15). In addition to the clear histopathological similarities, canine share the same risk factors for the onset of this disease, including obesity, hormones, and genetic alterations (16, 17). Additionally, the expression of several lineage-specific markers is identical in canine and human and immunohistochemically similar mammary tumor subtypes and mammary pre-neoplasias have been detected (15, 18, 19). These data support the usefulness of these spontaneous carcinomas as a model for studying specific breast cancer subtypes and in understanding the mechanisms of cancer cell invasion.

In this study, we characterized two distinct CMT cell lines, CMT-U27 and CMT-U309, in respect of their lineage-specific markers and actin cytoskeleton. Additionally, as these cells were displaying very different organization of actin-based structures, we wanted to test the possible interconnection between contractile forces and invasion in 3D environment. The studied cell lines belonged to different subcategories of mammary tumors: CMT-U27 representing a simple carcinoma and CMT-U309 representing a spindle cell carcinoma (20, 21). Simple carcinomas are composed of only one cell type, resembling either luminal epithelial or myoepithelial cells (22), while spindle cell carcinomas of the breast typically contain clusters of elongated cells, which resemble more mesenchymal than epithelial type of cells and are rare and aggressive subtypes of mammary tumors both in human and dog (23–25). As expected, analysis of these two cell lines with several lineage-specific markers revealed clear differences between the cell lines, showing that spindle carcinoma and simple carcinoma cells expressed predominantly basal or luminal cytokeratin markers, respectively. Of novel significance, CMT-U309 cells displayed greater number of mature actomyosin bundles and exerted significantly larger contractile forces in comparison with CMT-U27 cells. These high cell-mediated forces were also linked to the invasion potential of this cell line, while each of these effects were abolished by inhibition of 5′ AMP-activated protein kinase (AMPK) signaling. Our findings, therefore, implicate AMPK signaling as a key regulator of both actin-based structures and invasiveness in CMT-U309 cells and further supports the findings on the role of biophysical properties of the cells in cancer cell invasion.

## Materials and Methods

### Cell Culture

Canine mammary tumor-U27 and CMT-U309 CMT cell lines (20, 21) were a kind gift from Prof. Eva Hellmen at Uppsala University. CMT-U27 was isolated from simple carcinoma and CMT-U309 from spindle cell tumor. Cells were cultured in RPMI 1640 with 10% FBS and penicillin–streptomycin in an incubator with 5% CO_2_, at +37°C temperature and were plated on dishes 1 day prior to experiments.

### Immunofluorescence Microscopy

Cells were cultured on glass coverslips, washed with PBS and fixed with 4% PFA. Fixed cells were permeabilized with 0.1% Triton X-100 in TBS for 5′ and moved to 0.2% Dulbecco/BSA. Immunofluorescence stainings were performed as described previously (26) and were repeated at least three times. The following primary antibodies were used in stainings: anti-E-cadherin (#14472, Cell Signaling Technology, Inc., Danvers, MA, USA), anti-vimentin (#5741, Cell Signaling Technology, Inc., Danvers, MA, USA), and anti-vinculin antibody (1:50) (hVin-1, Sigma, Saint Louis, MO, USA). The following secondary antibodies were used to detect the primary antibodies: Alexa Fluor α-rabbit 488 and α-mouse 568 (Life Technologies™, Carlsbad, CA, USA). Other reagents: Alexa-488- and -647-Phalloidins were used to visualize actin cytoskeleton in 1:200 dilution (Life Technologies™, Carlsbad, CA, USA), DAPI for DNA (Life Technologies™, Carlsbad, CA, USA), and DABCO/Mowiol was used in mounting. The images were acquired with Leica DM6000 upright fluorescence wide field microscope equipped with Hamamatsu Orca-Flash4.0 V2 sCMOS camera.

### Western Blotting

Cells were washed with PBS and lysed in 1% Triton X-100/PBS with protease and phosphatase inhibitor cocktail sets (539131 and 539131, Calbiochem, San Diego, CA, USA). Protein concentrations were measured with Qubit^®^ Protein Assay Kit (ThermoFisher Scientific, Waltham, MA, USA). Sample loading buffer 4× LSB-DTT buffer was added to lysates and samples were boiled for 5′ before loading the samples in SDS-PAGE gels. Semidry transfer and Immobilon-P Membrane, PVDF filter (Millipore, Billerica, MA, USA) was used for blotting. Mixture of 5% milk/BSA was used for blocking. Following antibodies were used for detection of specific proteins: mouse anti-p63 (4A4, Abcam, Cambridge, UK), rabbit anti-slug (#9585, Cell Signaling Technology, Inc., Danvers, MA, USA), mouse anti-E-cadherin (#14472, Cell Signaling Technology, Inc., Danvers, MA, USA), rabbit anti-N-cadherin (13116, Cell Signaling Technology, Inc., Danvers, MA, USA), rabbit anti-claudin-1 (#13255, Cell Signaling Technology, Inc., Danvers, MA, USA), mouse anti-cytokeratin 5 (XM26, Abcam, Cambridge, UK), rabbit anti-cytokeratin 14 (EPR17350, Abcam, Cambridge, UK), anti-Pan-keratin mAb (C11, #4545, Cell Signaling Technology, Inc., Danvers, MA, USA), rabbit anti-vimentin (#5741, Cell Signaling Technology, Inc., Danvers, MA, USA), rabbit anti-P-Thr172-AMPK (#4188, Cell Signaling Technology, Inc., Danvers, MA, USA), anti-calponin 1/2/3 Antibody (FL-297, Santa Cruz, CA, USA), anti-tropomyosin 1, 2, 3 (TM31; T2780, Sigma, Saint Louis, MO, USA), rabbit anti-P-thr18/ser19-myosin light chain (MLC) (3674, Cell Signaling Technology, Inc., Danvers, MA, USA), mouse anti-β-actin (clone AC-15, Sigma, Saint Louis, MO, USA), mouse anti-smooth muscle actin (α-SMA, clone 1A4, A5228, Sigma, Saint Louis, MO, USA), mouse anti-GAPDH (G8795, Sigma Aldrich, Saint Louis, MO, USA). Anti-mouse or -rabbit HRP-linked secondary antibodies (Cell Signaling Technology, Inc., Danvers, MA, USA) and Western HRP substrate (Luminata™Crescendo, WBLUR0100, Millipore, Billerica, MA, USA) were used for chemiluminescence detection of the protein bands. All Western Blot experiments were repeated at least three times.

### Cell Proliferation Assays

Cell proliferation assays were performed with Cell IQ live-cell imaging setup as in wound-healing assays. For measuring cell doubling time, CMT-U309 and CMT-U27 cells were sparsely plated on 12-well plate 1 day before the actual experiment. The following day, cell growth/proliferation was monitored for 24 h and images were acquired every 30 min. Cell growth rate (GR) was calculated by using the formula: $\mathrm{GR}=\frac{\text{ln}(N(t)/N(0))}{t}$, and cell doubling time was calculated: $\frac{\text{ln}(2)}{\mathrm{GR}}$, where *N* (*t*) = number of cells in the end of experiment, *N*(0) is cell number in the beginning and *t* = time in hours.

### Contractile Function

Cells were cultured on fibronectin (FN)-coated, shape-determined micropatterns (CYTOOchips™, Grenoble, France) for 6 h and fixed with 4% PFA for 20′. Actin cytoskeleton was stained with Alexa-488-Phalloidin and nuclei with DAPI. Analysis of contractile function is based on the curvature of the formed thick contractile actomyosin bundle (see also Figure 3B). Radius of curvature (*R*) was calculated by using the formula: $R=\frac{{\left(L/2\right)}^{2}+w^{2}}{w}$, where L = length and w = width. Measurements of cell edges were done with ImageJ program.

### Traction Force Microscopy (TFM)

To measure cell-exerted traction forces, CMT-U27 and -U309 cells were cultured for 2–4 h on elastic collagen-1-coated polyacrylamide-based gel substrates of known stiffness (Young’s Modulus/elastic modulus = 11 kPa). Substrates were surface-coated with sulfate fluorescent microspheres (Invitrogen, Carlsbad, CA, USA, diameter 200 nm). Single isolated cells together with the underlying microspheres were imaged with 3I Marianas imaging system (3I intelligent Imaging Innovations, Germany), at multiple locations. To perform the imaging, a 63×/1.2 W C-Apochromat Corr WD = 0.28 M27 objective was used. The system was placed in a heated sample chamber (+37°C) and controlled for CO_2_. Following live cell imaging, the cells were detached from the substrates with 10 × Trypsin (Lonza Group, Basel, Switzerland) and a second set of microsphere images were obtained in a cell-free configuration. These images served as reference. Cell-exerted traction forces cause displacement of microspheres within the elastic substrate and spatial maps of microsphere displacements were achieved by comparing the reference microsphere images together with the experimental images. With knowledge of the cell-exerted displacement field, substrate stiffness (11 kPa), and a manual trace of the cell boundary, we computed the cell-exerted traction field using Fourier Transform Traction Cytometry (27, 28). From the traction field, we computed the root mean squared magnitude.

### Wound-Healing Assay

Wound-healing assays were performed to study cell migration by using Cell IQ live-cell imaging setup consisting of a microscope (Nikon) equipped with 10×/0.30 Ph1 objective and an incubator at +37°C with CO_2_. Images were captured with Qimaging Retiga EXi camera using Cell IQ Imagen software. For wound-healing assay, CMT-U309 and CMT-U27 cells were grown to confluency on 12-well plate (CellStar). The wound (gap) was created by scratching the confluent monolayer with a 200 µl pipette tip, and the debris was removed immediately by rinsing twice with DMEM. Cells were monitored for 24 h and images were acquired every 30 min. Data were analyzed using Cell IQ Analyser. The half-closure time was calculated by using the formula: $t1/2\text{gap}=\frac{\text{Initial gap Area}}{2\text{xslope}}$. Gap area was measured and plotted as a function of time. Cell migration rate (*v*_migration_, micrometer per hour) was calculated from the slope of this plot: $\text{υ migration}=\frac{\text{slope}}{2\text{xl}}$, where *v* = velocity and *l* = length of the gap.

### 3D Cell Culture

ECM gel from Engelbreth-Holm-Swarm murine sarcoma (Sigma E1270) was prepared according to manufacturer’s instructions. CMT-U309 and CMT-U27 cells were trypsinized and ~5,000 cells in DMEM were seeded on top of the matrigel-coated eight-chamber slides. 3D spheroids were observed under microscope daily for 2 weeks.

### Manipulation of AMPK Activity

For the inhibition of AMPK activity, cells were treated with AMPK inhibitor, compound C (Sigma-Aldrich). On 2D cultures, final concentration of compound C was 5 μM and incubation time 3–6 h.

In 3D spheroid cultures, 15 µM compound C was applied on cells for 6 days to completely inhibit AMPK activity. For the activation of AMPK activity, cells were treated with 25 μM AICAR (Sigma Aldrich, Saint Louis, MO, USA) for 16 h.

### Statistical Analyses and Data Presentation

SDs and statistically significant difference between means of two groups (*t*-test) were analyzed in Microsoft Excel 2013. Box charts were done with OriginPro 8.6 program (Whisker range 5–95, showing outliers) and column charts were done with Microsoft Excel 2013.

## Results

### CMT-U27 and CMT-U309 Cell Lines Exhibit Distinct Morphological Features and Differential Expression of Lineage-Specific Markers

**Figure 1:**
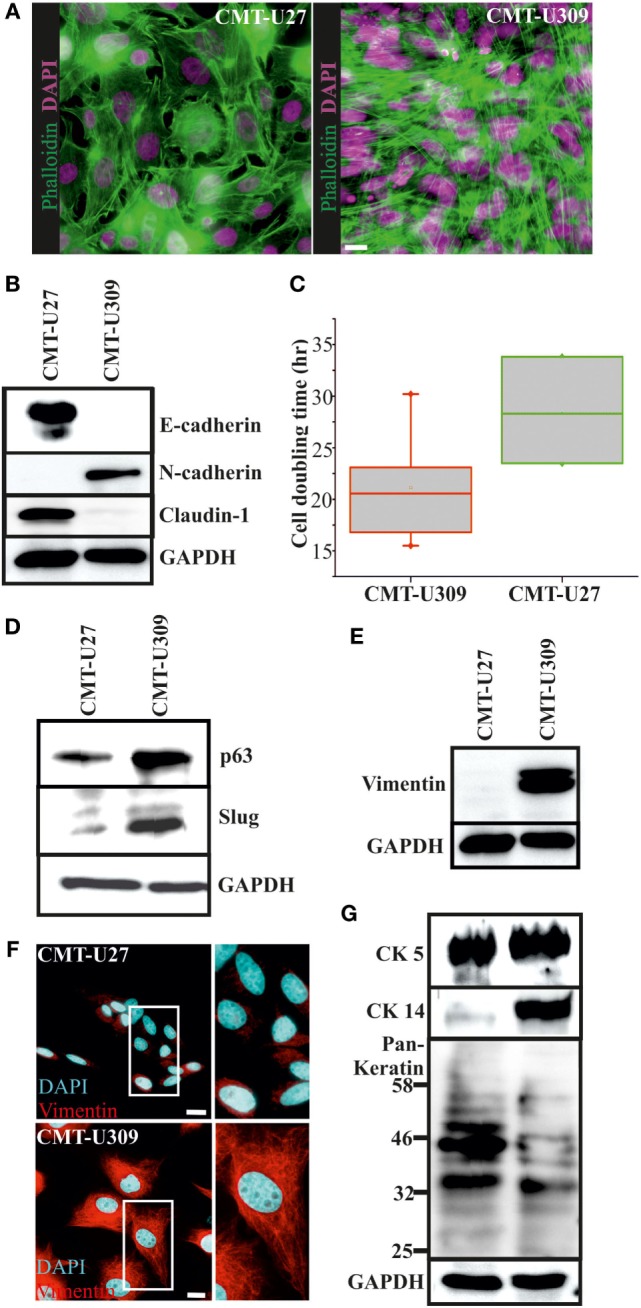
Morphology and expression of lineage-specific markers in canine mammary tumor (CMT)-U27 and -U309 cell lines. **(A)** CMT-U27 and -U309 cells exhibit distinct morphological features, U27 cells had more variation in cell size and shape, while U309 were mostly elongated and typically not contact inhibited by the confluency. Actin cytoskeleton is stained with phalloidin and nuclei with DAPI. Scale bar 10 μm. **(B)** Western Blotting from cellular lysates revealed that epithelial cell–cell contact proteins E-cadherin and claudin-1 are absent from CMT-U309 cells, while these cell express N-cadherin. In contrast, CMT-U27 cells possess expression of both E-cadherin and claudin-1, typical for luminal epithelial cells. Western Blot experiments were repeated at least three times. **(C)** Proliferation rates and average cell doubling times of CMT-U27 and CMT-U309 cell lines were measured from sparsely growing cultures in an o/n experiment (see Materials and Methods for details). Values are presented in box-plots, where the median is indicated by the central bar. 5/95 percentile whiskers with outliers are shown; *n* = 3–6. **(D)** Western blot experiments from the cellular lysates of CMT-U27 and -U309 cell lines showed that CMT-U309 cells express both p63 and slug, typical markers for the basal breast epithelial cells, while CMT-U27 cells possess almost non-detectable levels of these proteins. Western Blot experiments were repeated at least three times. **(E)** Mesenchymal marker vimentin is highly expressed in CMT-U309 cells as shown by western blots from the cellular lysates of CMT-U27 and -U309 cells as well as immunofluorescence stainings with anti-vimentin antibody **(F)**. Nuclei are visualized with DAPI. Scale bar 10 μm. **(G)** Expression of cytokeratins was studied with anti-CK5 anti-CK14 and anti-Pan-Keratin antibody recognizing CK4, 5, 6, 8, 10, 13, 18 by Western Blotting from cellular lysates. Expression of CK5 was detected in both studied cell lines, while CK14 was detected only in CMT-U309 cells. Detection with Pan-Keratin ab showed differences in the expression of CK8 and 18 (53 and 48 kDa, respectively) that are typical for the luminal epithelial cells. Western Blot experiments were repeated at least three times.

We examined the morphological features and a set of lineage-specific markers of the two established CMT cell lines, simple carcinoma CMT-U27, and spindle cell carcinoma CMT-U309 (20, 21). Some of these markers have previously been shown by other studies (20, 21, 29). CMT-U27 exhibited pleomorphic cells as shown in Figure 1A, left panel. These cells were contact inhibited by increasing cell confluency, while the spindle carcinoma cells, CMT-U309, had an elongated-morphology and tended to overgrow the neighboring cells (Figure 1A, right panel). The examination of epithelial cell adhesion proteins showed that E-cadherin and claudin-1 are not expressed in CMT-U309 cells (Figure 1B). In contrast, CMT-U309 cells were showing high levels of N-cadherin (Figure 1B), a protein, which is often associated with the decrease in E-cadherin expression in so-called *cadherin switch* during epithelial-to-mesenchymal transition and linked to metastatic breast cancer (30, 31). CMT-U27 cells did not show any expression of N-cadherin but had clear expression of epithelial adhesion proteins E-cadherin and claudin-1 (Figure 1B).

The average cell doubling time for CMT-U27 and CMT-U309 was found to be 28.5 and 21.1 h, respectively (Figure 1C). Faster proliferating spindle carcinoma CMT-U309 cells showed clear reactivity for the basal cell marker p63, while CMT-U27 cells expressed very low levels of this marker (Figure 1D). Transcription factor p63 has been shown to be highly expressed in both human and canine spindle cell mammary cancer tumors (32, 33). CMT-U309 cells also expressed snail family transcription repressor slug that has been implicated in maintaining the basal phenotype [reviewed in Ref. (34)], while CMT-U27 cell line had almost non-detectable levels of this protein (Figure 1D). Furthermore, CMT-U27 cells did not show any reactivity for the mesenchymal marker vimentin, while it was highly expressed in CMT-U309 spindle cells (Figures 1E,F).

Cytokeratins (CKs) play a significant role in the progression of mammary tumors (35). Therefore, we examined the expression of CKs 4, 5, 6, 8, 10, 13, 14, and 18 by utilizing a Pan-Keratin antibody as well as by using a specific antibody against CK5 and CK14. Both CMT-U27 and CMT-U309 showed similar expression of CK5, as detected by western blotting (Figure 1G). Basal CK14 was distinctly expressed in the spindle carcinoma cells (Figure 1G). The western blot analyses with Pan-Keratin antibody showed similar pattern of protein bands between these cell lines. However, the intensity of bands corresponding to the size of luminal epithelial markers CK8 and CK18 (53 and 48 kDa, respectively), showed clearly lower levels in CMT-U309 cell line (Figure 1G). Thus, the expression patterns, verified by several lineage-specific markers, were suggestive for distinct luminal- and basal-like features within the studied cell lines.

### Organization and Expression of Actin Cytoskeleton-Associated Proteins in CMT-U27 and -U309 Cell Lines

**Figure 2:**
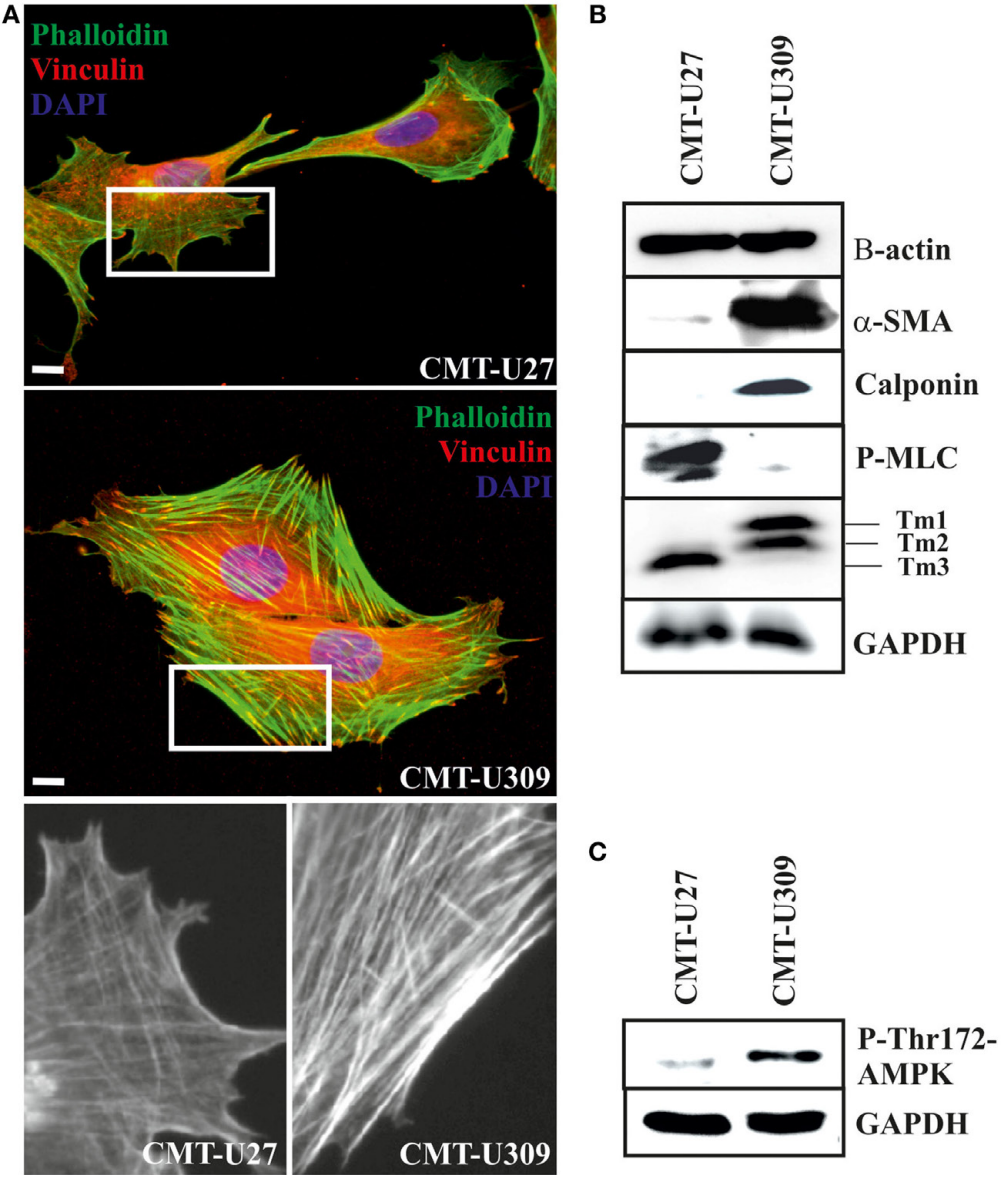
Canine mammary tumor (CMT)-U27 and CMT-U309 cells display differential organization of actin cytoskeleton and expression of actin-associated proteins. **(A)** Simple carcinoma cells CMT-U27 possess both thick contractile actomyosin bundles as well as thinner actin-based structures both perpendicular and parallel to the leading edge of the cells. Spindle carcinoma cells, CMT-U309, have predominantly thick straight actomyosin bundles that are connected to focal adhesion sites from their both ends. Magnification of the cell edges of both cell lines are shown below. Phalloidin-green, focal adhesion marker vinculin-red and DAPI-blue. Scale 10 μm. **(B)** Western blot analyses of cellular lysates from the studied cell lines showed that both cell lines express high levels of β-actin, while high expression of smooth muscle actin, α-SMA, was detected only in spindle cell carcinoma U309. These cells were devoid of non-muscle myosin as detected with the phospho-thr18/ser19-myosin light chain (MLC) antibody. Differential expression of calponin-1 and specific tropomyosin isoforms was also detected between these cell lines: CMT-U309 cells expressed high levels of calponin-1, typical for basal cells, while this protein was not detected from the lysates of CMT-U27 cells. CMT-U27 expressed mainly tropomyosin isoform, Tm3, while CMT-U309 cells expressed Tm1 and Tm2. Western Blot experiments were repeated at least three times. **(C)** Expression of active form of AMP-activated protein kinase (AMPK) kinase, phospho-Thr172-AMPK, was analysed from the cell lines as it has been linked to maturation of thick contractile actomyosin bundles (26). High levels of P-Thr172-AMPK was detected from the lysates of CMT-U309 cells, correlating with the appearance of thick actomyosin bundles in these cells.

Actin cytoskeleton undergoes dramatic reorganization during the process of malignant transformation (36). During this transformation, several actin-binding proteins are known to be deregulated and contribute to the development of abnormal adhesive and migratory properties of the cancer cells. We examined the organization of actin cytoskeleton as well as expression of cytoskeletal proteins in CMT-U27 and CMT-U309 cancer cell lines (Figure 2A). CMT-U27 cells exhibited variable organization of their actin cytoskeleton, including both thick contractile bundles as well as thin actin-based structures both parallel and perpendicular to the leading edge of the cells (Figure 2A). These thin structures resembled structures that act as precursors for the mature thick contractile actomyosin bundles (26, 37, 38). In contrast, CMT-U309 cells had very prominent, thick contractile actin-based bundles, bound to focal adhesion sites from their both ends (Figure 2A).

Both CMT-U27 simple carcinoma and CMT-U309 spindle carcinoma cells appeared to have similar expression level of β-actin (Figure 2B). Additionally, spindle carcinoma cells expressed high levels of smooth muscle actin (α-SMA) and calponin-1 (Figure 2B), typical for these type of carcinoma cells. In respect of non-muscle myosin and high molecular weight tropomyosins (Tm1, 2, and 3), these cell lines showed also clear differences. CMT-U27 possessed high levels of phosphorylated non-muscle MLC (P-thr18/ser19-P-MLC), indicative of active myosin that is required for the contraction. In contrast, in CMT-U309, its level was almost non-detectable (Figure 2B). These cell lines had also distinct expression patterns of tropomyosins as CMT-U309 showed predominantly expression of Tm1 and Tm2, while CMT-U27 had high expression of Tm3 isoform (Figure 2B).

As the maturation of contractile actomyosin bundles from their precursor was recently found to be dependent on the activity of 5′ AMPK (26), we examined the levels of active AMPK, P-Thr-172 AMPK, from the lysates of both CMT cell lines (Figure 2C). Lysates from CMT-U27 cells contained low levels of active AMPK. In contrast, CMT-U309 cells were found to have high levels of P-thr172-AMPK, correlating well with the presence of thick, mature actin-based bundles in these cells. The results suggest that the high AMPK kinase activity could be responsible for the great number of mature actomyosin bundles in CMT-U309 cells.

### CMT-U309 Cells Display High Contractile Forces

**Figure 3:**
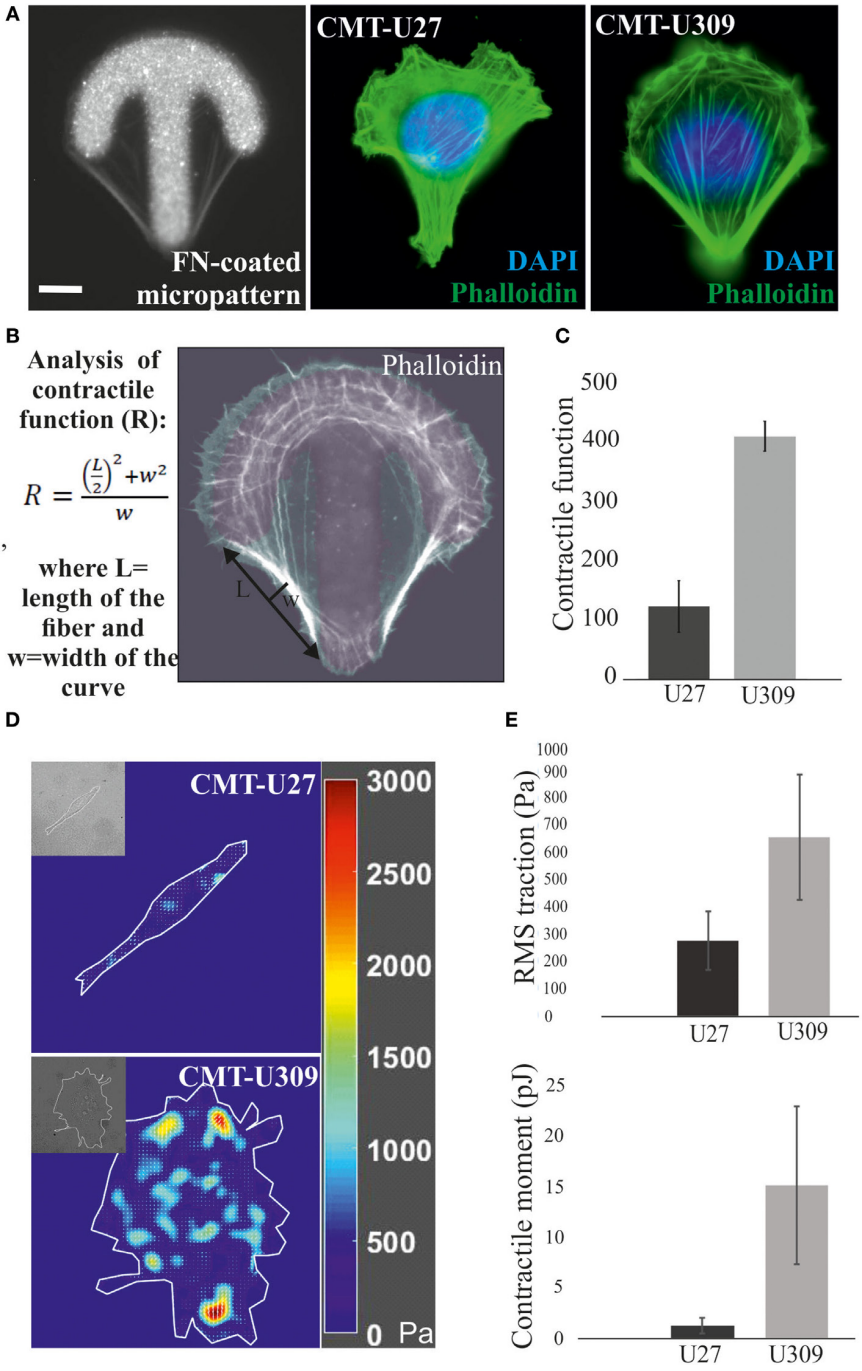
Analysis of contractile function and cell-mediated forces. **(A)** Canine mammary tumor (CMT)-U27 and CMT-U309 cells were cultured on shape-determined, fibronectin (FN)-coated micropatterns (CYTOOchips™), and visualized for the organization of actin cytoskeleton. CMT-U309 cells displayed again only thick straight bundles, while CMT-U27 cells had thinner structures with more variability in their organization pattern. Phalloidin-green, DAPI-blue. Scale bar 10 μm. **(B)** Contractile function of the actin-based structures was analyzed from the cells cultured on these micropatterns. Contractility is directly correlated with the radius of curvature and is calculated by using the formula: $R=\frac{{\left(L/2\right)}^{2}+w^{2}}{w}$, where *L* = length and *w* = width. **(C)** Mean contractile function of CMT-U27 and CMT-U309 cells ± SD, *n* = 32 (U27), *n* = 39 (U309). **(D)** Representative examples of traction force maps of CMT-U27 and CMT-U309 cells. Unit of stress is in pascals (Pa). **(E)** Root mean square tractions of CMT-U27 and CMT-U309 cells are shown in the upper graph and contractile moment in the lower graph. Means ± SD are shown (*n* = 7 for U27; *n* = 10 for U309).

Actin-based structures provide force for many cellular processes [reviewed in Ref. (39)]. These contractile actomyosin forces can be exerted through the adhesion sites to the surrounding extracellular matrix as traction forces. We analyzed the contractile properties of CMT-U27 and CMT-U309 cell lines by utilizing FN-coated shape-determined micropatterns on 2D cultures (CYTOOchips™) (Figures 3A,B). Cells cultured on these patterns organize their mature contractile bundles at the edges of these patterns and precursor structures are typically found behind the formed leading edge of the cell, at the arc of the micropattern (Figure 3A). The curvature of the formed contractile bundle is related to its contractility. Contractile function of the cells, cultured on these patterns, can be calculated as radius of curvature, *R*, as described in Figure 3B. Based on this analysis method, CMT-U27 cells were significantly less contractile than CMT-U309 cells (Figure 3C).

Furthermore, we measured the cell-exerted forces directly by utilizing TFM. In this method, cells are cultured on an elastic substrate, embedded with fluorescent microspheres, and the cell-exerted forces cause displacement of these beads. With the knowledge of the microsphere displacement field and stiffness of the elastic substrate, cell-exerted traction forces can be computed. Consistent with the contractility measurements by using micropatterns, described above, CMT-U27 exerted significantly smaller traction forces than CMT-U309 cells (Figures 3D,E).

### Invasion Potential of CMT-U309 Is Dependent on Its High Traction Forces

**Figure 4:**
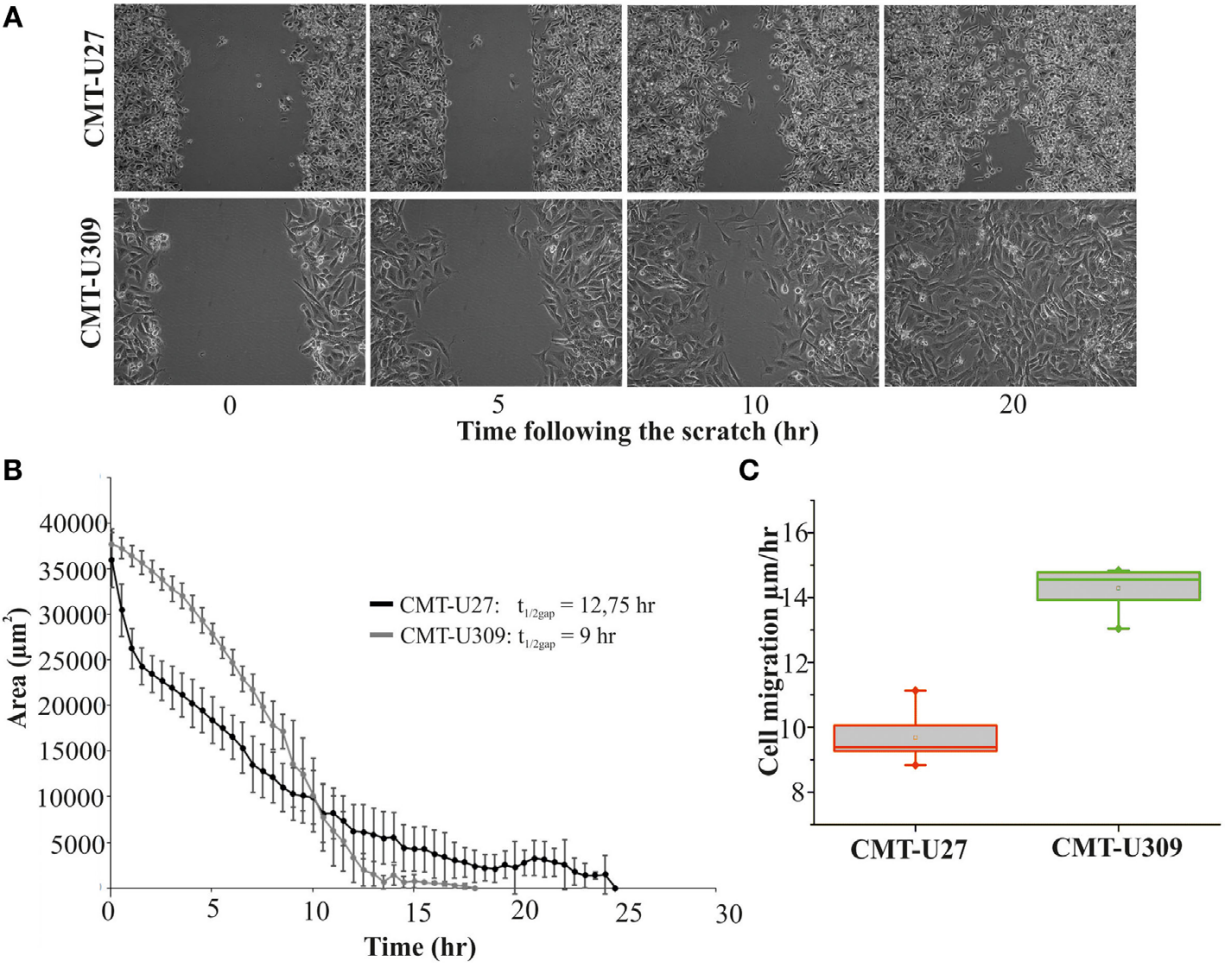
Migration speed of canine mammary tumor (CMT)-U27 and CMT-U309 on 2D environment. **(A)** Images from a wound-healing assay at different time points. CMT-U27 and CMT-U309 cells were grown to confluency, wounded with a pipette tip, and then imagined 24 h with automated cell IQ live-imaging platform. **(B)** Wound area recovery over time. The gap area was quantified for each time frame and for each cell line using Cell IQ Analyser. The half-closure times were calculated by using the formula: $t\text{1/2gap}=\frac{\text{Initial gap Area}}{\text{2xslope}}$. Mean ± SD is shown, *n*(U27) = 6 and *n*(U309) = 8. **(C)** Average migration speed (micrometers per hour) of CMT-U27 and CMT-U309 cell lines. Migration speed was calculated: $\upsilon \;\text{migration}=\frac{\text{slope}}{\text{2xl}}$. Values are presented in box-plots, where the median is indicated by the central bar. 5/95 percentile whiskers with outliers are shown; *n*(U27) = 6 and *n*(U309) = 8.

**Figure 5:**
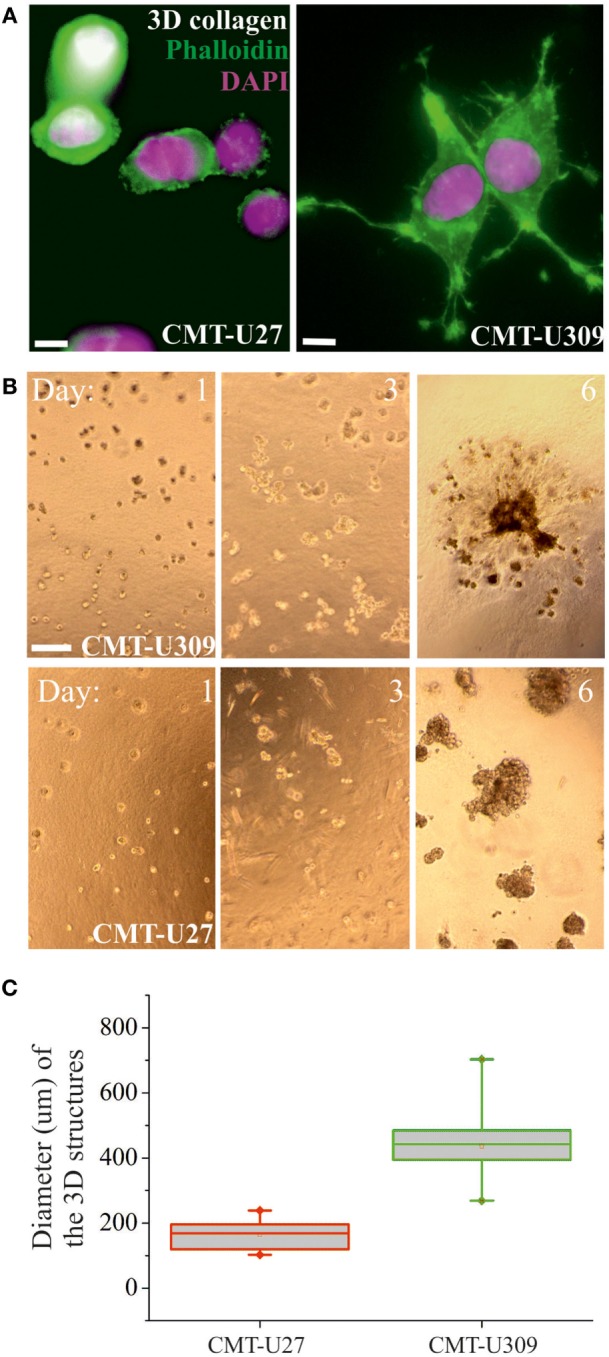
3D morphology and invasion potential of canine mammary tumor (CMT)-U27 and CMT-U309 cell lines. **(A)** Confocal images of CMT-U309 and CMT-U27 in 3D collagen matrix. CMT-U27 cells adopt round and blebbing conformation, while CMU309 cells exert long actin-based protrusions. Phalloidin-green, DAPI-pink. Scale bar 10 μm. **(B)** CMT cell lines have different invasion potential in 3D matrigel cultures. Brightfield images of cultures taken on days 1, 3, and 6 are shown. Both CMT-U27 and CMT-U309 cell lines form initially spheroid-like structures, which start to expand rapidly. CMT-U27 cells have deficient growth control and form very dense structures but do not invade, while CMT-U309 cells clearly start to dissociate from the original 3D spheroid and form secondary structures outside. Scale 100 μm. **(C)** Quantification of the diameter of the expanding cellular 3D structure 6 days after culture. Values are presented in box-plots, where the median is indicated by the central bar. 5/95 percentile whiskers with outliers are shown; *n* = 15.

Recent studies have shown that cell-substrate forces directly correlate with the invasion potential of cancer cells in 3D matrices and that several invasive human cancer cell lines apply higher forces than their non-invasive counterparts (7, 8). We compared the migratory properties of CMT-U27 and CMT-U309 in both 2D cultures and in 3D matrigel cultures. The migration speed of CMT-U27 and CMT-U309 cells was 9.5 and 14.5 μm/h, respectively (see Figures 4A–C). Confocal images showed that, in 3D collagen cultures, the morphology of these two cell types were totally different (Figure 5A): CMT-U27 cells were rounded with cortical actin staining and bleb formation, while CMT-U309 cells exhibited long actin-based protrusion and elongated form. In 3D matrigel cultures, both cell lines initially formed round symmetrical spheroids (Figure 5B). After 3 days in culture, the spindle carcinoma cells started to exhibit invasive features and escape from the primary structures that had already undergone through massive expansion (Figures 5B,C). CMT-U27 cells were highly proliferative but did not invade to the surrounding matrix, while CMT-U309 cultures showed single invading cells that started to form new spheroids outside the primary structures (Figure 5B).

**Figure 6:**
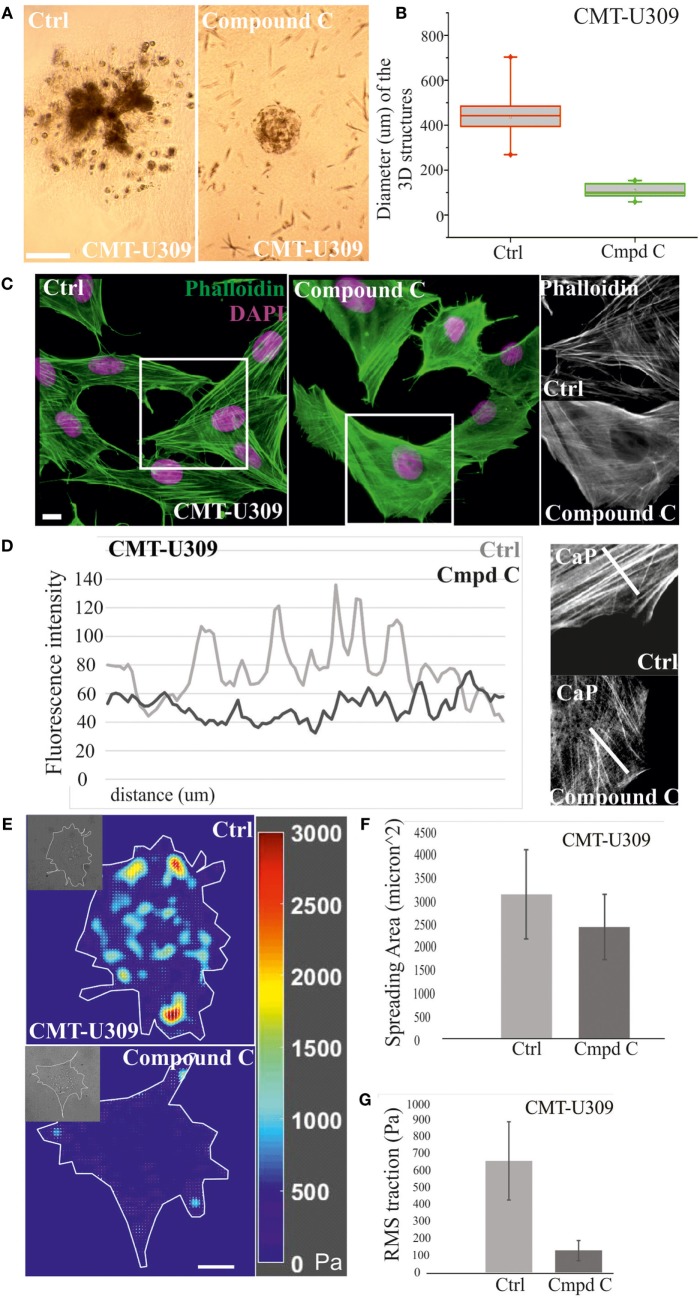
Chemical inhibition of AMP-activated protein kinase (AMPK) activity in canine mammary tumor (CMT)-U309 cells blocks the invasion potential of these cells. **(A)** CMT-U309 cells were cultured in 3D matrigel either in the presence or absence of AMPK inhibitor compound C for 14 days. Brightfield images taken on day 8 are shown. Cells incubated with the chemical compound were still forming symmetrical 3D scaffolds but their invasion capability was totally inhibited. Scale bar 100 μm. **(B)** Quantification of the diameter of the formed 3D structures in matrigel between days 6 and 8. Data are presented in box-plots, where the median is indicated by the central bar. 5/95 percentile whiskers with outliers are shown; *n* = 17 for ctrl and *n* = 15 for compound C-treated cultures. **(C)** Treatment of CMT-U309 cells with compound C leads to decrease in the amount of thick contractile actomyosin bundles on 2D cultures as visualized by phalloidin staining. Scale bar 10 μm, DAPI is visualized with pink color, actin-green. **(D)** Lineprofiles of ctrl and compound C-treated CMT-U309 cells show decreased intensity and width of the fibers, indicating appearance of thinner actin-based structures in AMPK-inhibited cells. Lineprofiles were drawn 20 μm perpendicular from the edges of the cells as shown on the right side panels. Fluorescence intensities along the lineprofiles were measured with ImageJ. **(E)** Treatment of CMT-U309 cells with compound C leads to decreased cell-mediated traction forces. Images of representative cells and the corresponding force maps are shown. **(F)** Quantification of the spreading area and average root mean square traction forces **(G)** of untreated or AMPK inhibitor-treated cells. AMPK inhibition led to small decrease in cell size, while the difference in cell-mediated forces was significant, AMPK-inhibited cells displaying only about 20% of the original forces. Results are shown as ±SD (*n* = 10, U309 ctrl cells; *n* = 6, U309 compound C-treated cells).

Next, we examined whether the invasion potential of CMT-U309 cell line was dependent on its contractile properties. Since the maturation of force-producing actomyosin bundles was recently shown to be controlled by AMPK (26) and CMT-U309 cells also exhibited high levels of active AMPK (Figure 2C), we tested how inhibition of this kinase would affect contractility and actin-based invasion in this particular cell line. AMPK activity was inhibited by compound C in 3D matrigel cultures (Figures 6A,B). Importantly, in the inhibitor-treated 3D cultures, CMT-U309 cells still proliferated and formed predominantly symmetrical spheroid-like structures. However, their ability to invade was completely prevented (Figure 6A). To verify that AMPK inhibition actually affects the formation and morphology of contractile actomyosin bundles, we performed 2D cultures of these cells and analyzed the organization of actin cytoskeleton in both ctrl and inhibitor-treated cells. Inhibition of AMPK activity for 6 h was found to significantly decrease the amount of fully matured contractile actomyosin bundles and in contrast increase the amount of thinner precursor structures (Figures 6C,D). In line with this, opposite stress fiber phenotype was detected in CMT-U27 cells that were exposed to a commonly used AMPK activator, AICAR (Figure S1 in Supplementary Material), suggesting that the maturation of contractile actomyosin bundles is dependent on AMPK activity also in these particular CMT cell lines. Furthermore, we analyzed whether the effect of AMPK inhibitor on the organization of contractile actomyosin bundles in CMT-U309 cells could be dependent on the expression levels of α-SMA, calponin-1, and tropomyosins. However, we could not detect any alterations in their levels in Western Blots within the same time frame as the reorganization of actin-based structures was detected (data not shown).

Moreover, we wanted to analyze how these changes in actin-based structures after AMPK inhibition would be reflected to cell traction forces. TFM was performed in ctrl (non-treated) and compound C-treated CMT-U309 cells (Figure 6E). After the inhibition, the cell spreading area was about 80% of the size of untreated cells (Figure 6F), while the average Root Mean Square tractions in AMPK-inhibitor-treated cells were approximately five times smaller in comparison with ctrl cells (Figure 6G). The decrease in cell-mediated forces correlated well with the detected decrease in the amount of thick actomyosin bundles after AMPK inhibition (Figures 6C,D). Our results suggest that AMPK activity controls the maturation of force-producing actomyosin bundles in this cell line and that high traction forces are connected to the invasion potential of these canine spindle carcinoma cells.

## Discussion

Canine mammary tumors provide a more natural model system to study the complex biology of human breast carcinomas in comparison with the induced rodent tumors (15, 40, 41). In this study, we utilized two different subtypes of CMT cell lines, simple carcinoma CMT-U27 and spindle cell carcinoma CMT-U309, to characterize their subtype-specific differences in the expression of lineage-specific markers as well as to clarify the link between actomyosin forces and invasion potential of cancer cells within 3D environment. We found that CMT-U27 and CMT-U309 cell lines exhibited distinct expression patterns toward luminal and basal markers, respectively (Figures 1 and 2). Basal markers α-smooth muscle actin, calponin, vimentin, and p63 that were expressed by CMT-U309 have been mostly associated with poor prognosis in human breast cancers (42, 43). Of the other basal markers, only cytokeratin 5 (CK5) was expressed in both cell lines. CK5 is typical for progenitors and intermediary cells in both human and canine and approximately 75% of the canine simple carcinoma cases are known to express this cytokeratin isoform (44, 45). Otherwise, the cytokeratin expression pattern was different in between the cell lines, CMT-U309 expressing CK14 and CMT-U27 more luminal epithelial CKs (Figure 1G). Expression of epithelial CKs CK8, 18 and 19 are often lost during neoplastic transformation and expression of basal CKs as CK5/6, 14, and 17 start to dominate (46, 47). This kind of shift toward basal cytokeratin expression has been linked with an aggressive cancer phenotype in both canine and human (32, 35). In our study, the CMT-U309 cells with basal characteristics also appeared more aggressive in 3D culture setup than the luminal type CMT-U27 cells. Additionally, these CMT-U309 cells expressed high levels of Tm1, which in humans is associated with malignant conversion of mammary epithelium and resistance to anoikis (48).

What is then the origin of these invasive CMT-U309 cells? Spindle cell carcinoma of the breast represents a sub group of metaplastic carcinomas, showing variability in cellular composition and features (25). There has been discrepancy about the genetic origin of these types of breast carcinomas, and some studies have proposed them to be originated from myoepithelial cells (24) and some from luminal cells (49). Interestingly, the study by Hellmen et al. (21) showed that CMT-U309 spindle carcinoma cells possess plasticity and that they can give rise to phenotypically distinct tumor types *in vivo* (21). This suggests that CMT-U309 CMT line could be derived from pluripotent stem cells. In line with that, we found that CMT-U309 cell line expressed high levels of slug (Figure 1D), a member of the snail family transcription repressors, which is associated to the maintenance of the mammary stem cell-like state in both human and mouse (34, 50). Additionally, we could not detect any claudin-1 in CMT-U309 cells (Figure 1B) and claudin-1 has been shown to be repressed by slug (51, 52).

Spindle cell carcinomas are aggressive subtypes of mammary tumors and there are no standard treatment protocols (53). Canine spindle carcinoma cells, CMT-U309, possessed high contractility and invasion potential in 3D matrigel cultures, and these features were found to be dependent on high AMPK activity (Figures 3, 5 and 6). AMPK kinase has mainly been linked to the regulation of main metabolic pathways but it also contributes to other signaling and growth control routes (54). Due to its involvement in many cellular tasks, the role of AMPK in cancer progression has been controversial and is probably dependent on the cellular context. In breast cancers, this may potentially be dependent on the estrogen receptor status as estrogen has also been linked to the regulation of AMPK activity (55, 56). In CMT-U309 cells, high AMPK activity was clearly linked to the capability to invade (Figure 6A). Additionally, high AMPK activity was directly linked with the morphology of thick actin-based structures (Figures 2, 3 and 6). Chemical inhibition of AMPK activity led to a decrease in the amount of mature actomyosin bundles and increase in the number of thin precursor structures (Figures 6C,D). These findings are in line with the previous data on the role of AMPK in the maturation of muscle sarcomeres and non-muscle actomyosin bundles (26, 57). The change in the morphology of actomyosin structures in AMPK-inhibited cells was also directly reflected to the cell traction forces (Figures 3 and 6E,F). As manipulation of AMPK activity was not affecting the expression level of either α-SMA or other actin-associated factors (results not shown), the data indicate that changes in cell-mediated forces upon AMPK inhibition are observed due to disturbed maturation of actomyosin bundles.

In conclusion, this work shows that high actomyosin-mediated contractility and traction forces of CMT-U309 cell line were associated with its invasion potential and regulated by AMPK (Figure 6). Such a relationship, i.e., a link between high traction forces and cancer cell invasion, has been reported for many human cancer cell lines (4, 7, 58). Exertion of high traction forces may be predictive of the proteolytic degradation of ECM by invadopodia and, in this way, promote invasive phenotype (59). The data support the previous notions on the importance of cellular forces in neoplastic progression and cancer cell invasion.

## Author Contributions

KR has performed most of the experiments and analyzed data, RK has analyzed traction force data, and ST has performed imaging and written the manuscript.

## Conflict of Interest Statement

The authors declare that the research was conducted in the absence of any commercial or financial relationships that could be construed as a potential conflict of interest.

## Supplementary Material

Figure S1 AMP-activated protein kinase activation by chemical compound, AICAR, induces maturation of contractile actomyosin bundles in canine mammary tumor (CMT)-U27 cells. **(A)** CMT-U27 simple carcinoma cells were either left untreated or were treated with AICAR for 16 h. After fixation, cells were stained with Phalloidin-488 and DAPI to visualize cytoskeletal structures and nuclei. AICAR-treated cells showed clearly greater number of thick contractile bundles, bound to focal adhesion sites from their ends (white arrows), while ctrl cells displayed both precursor structures and mature bundles. Precursor network behind the leading edge of the cells is indicated with white brackets. Scale 10 μm. **(B)** Cells exhibiting only mature, force-producing structures were quantified from the imaging data. 26.5% of the ctrl cells possessed only mature actomyosin bundles, while after AICAR-treatment the amount of these cells had increased to 71.9%. *n*(ctrl cells) = 94 and *n*(AICAR cells) = 114.
