# Supplementary figures and images for: Analysis of Contractility and Invasion Potential of Two Canine Mammary Tumor Cell Lines
Source: Front Vet Sci. 2017 Sep 12;4:149. doi: 10.3389/fvets.2017.00149 (PMC5600937; doi:10.3389/fvets.2017.00149)

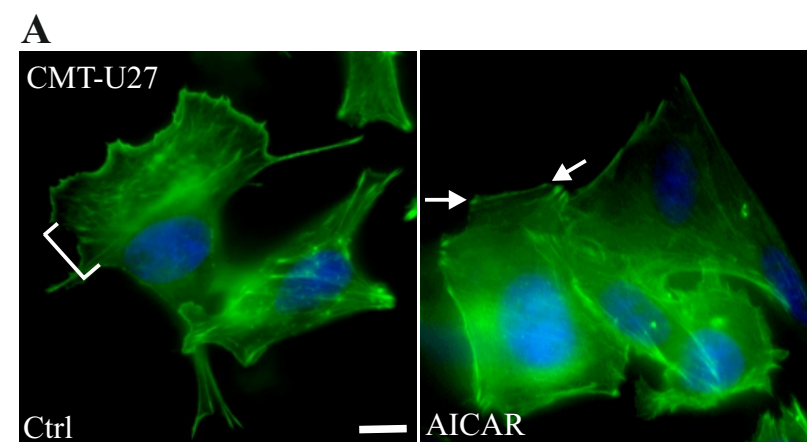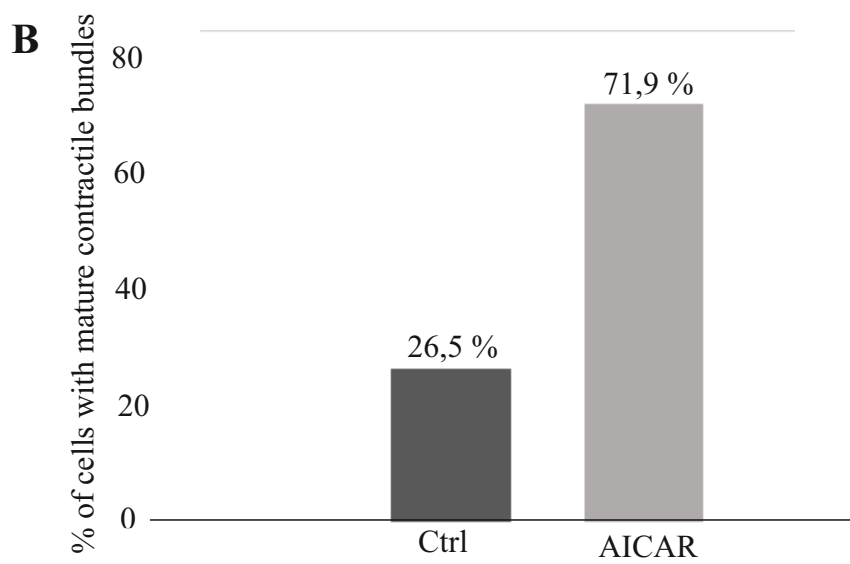

Rajakylä et al., Supplementary Figure 1

Supplement: Figure S1 — AMP-activated protein kinase activation by chemical compound, AICAR, induces maturation of contractile actomyosin bundles in canine mammary tumor (CMT)-U27 cells. (A) CMT-U27 simple carcinoma cells were either left untreated or were treated with AICAR for 16 h. After fixation, cells were stained with Phalloidin-488 and DAPI to visualize cytoskeletal structures and nuclei. AICAR-treated cells showed clearly greater number of thick contractile bundles, bound to focal adhesion sites from their ends (white arrows), while ctrl cells displayed both precursor structures and mature bundles. Precursor network behind the leading edge of the cells is indicated with white brackets. Scale 10 μm. (B) Cells exhibiting only mature, force-producing structures were quantified from the imaging data. 26.5% of the ctrl cells possessed only mature actomyosin bundles, while after AICAR-treatment the amount of these cells had increased to 71.9%. n(ctrl cells) = 94 and n(AICAR cells) = 114. [file image_1.pdf]
